# Battle of the Bonds: Training’s Impact on Dental Adhesive Use in Undergraduate and Continuing Education

**DOI:** 10.3290/j.jad.c_2433

**Published:** 2025-12-17

**Authors:** Sophie Gosselin, Lenny Dahan, Frederic Raux, Stephane Le-Goff, Sarah Abdel-Gawad, Helene Gouze, Maria-Antonietta D’Agostino, Timothy Fasham, Elisabeth Dursun, Emmanuel Bourdageau, Yasmine Smail, Jean-Pierre Attal, Mathieu A. Derbanne, Philippe François

**Affiliations:** a Sophie Gosselin Dentist, Department of Biomaterials, UMR 1333 Oral Health, University of Paris Cité, Bretonneau Hospital, Assistance Publique–Hôpitaux de Paris (AP-HP), 1 rue Maurice Arnoux, 92120 Montrouge, France. Concept and design; acquisition, analysis, and interpretation of data; writing: original draft preparation; writing: review and editing; read and approved the final manuscript.; b Lenny Dahan Dental Student, Department of Biomaterials, University of Paris Cité, Bretonneau Hospital, Assistance Publique–Hôpitaux de Paris (AP-HP), 1 rue Maurice Arnoux, 92120 Montrouge, France. Concept and design; acquisition, analysis, and interpretation of data; writing: original draft preparation; writing: review and editing; read and approved the final manuscript.; c Frederic Raux Dentist, President of the Académie De Dentisterie Adhésive (ADDA), Private Practice, 16 Avenue Pierre 1er de Serbie, 75016 Paris,France. Writing: review and editing; read and approved the final manuscript.; d Stephane Le-Goff Assistant Engineer, Department of Biomaterials, UMR 1333 Oral Health, University of Paris Cité, ADDA member, 1 rue Maurice Arnoux, 92120 Montrouge, France. Concept and design; writing: review and editing; read and approved the final manuscript.; e Sarah Abdel-Gawad EMSE, Department of Biomaterials, UMR 1333 Oral Health, University of Paris Cité, 1 rue Maurice Arnoux, 92120 Montrouge, France. Concept and design; writing: review and editing; read and approved the final manuscript.; f Helene Gouze Medical Doctor, Department of Rheumatology, CESP-INSERM, Catholic University of Sacred Health, Foundation Policlinico Universitario Agostino Gemelli IRCSS, Rome, Italy. Writing: review and editing; statistical analyses; read and approved the final manuscript.; g Maria-Antonietta D’Agostino Professor and Head of Department, Department of Rheumatology, Catholic University of Sacred Health, Foundation Policlinico Universitario Agostino Gemelli IRCSS, Rome, Italy. Writing: review and editing; read and approved the final manuscript.; h Timothy Fasham Assistant Professor, Department of Prosthodontics, UMR 1333 Oral Health, University of Paris Cité, Charles Foix Hospital, Assistance Publique–Hôpitaux de Paris (AP-HP), 1 rue Maurice Arnoux, 92120 Montrouge, France. Writing: review and editing; read and approved the final manuscript.; i Elisabeth Dursun Professor and Head of Department, Department of Pediatric Dentistry, UMR 1333 Oral Health, University of Paris Cité, Henri Mondor Hospital, Assistance Publique–Hôpitaux de Paris (AP-HP), 1 rue Maurice Arnoux, 92120 Montrouge, France. Writing: review and editing; read and approved the final manuscript.; j Emmanuel Bourdageau Scientific Marketing Manager, Kuraray Noritake French Division, 63 avenue du Général Leclerc, 92340 Bourg-la-Reine, France. Concept and design; writing: review and editing; read and approved the final manuscript.; k Yasmine Smail Assistant Professor, Department of Biomaterials, UMR 1333 Oral Health, University of Paris Cité, Bretonneau Hospital, Assistance Publique–Hôpitaux de Paris (AP-HP), 1 rue Maurice Arnoux, 92120 Montrouge, France. Writing: review and editing; read and approved the final manuscript.; l Jean-Pierre Attal Associate Professor and Chair, Department of Biomaterials, UMR 1333 Oral Health, University of Paris Cité, Charles Foix Hospital, Assistance Publique–Hôpitaux de Paris (AP-HP), 1 rue Maurice Arnoux, 92120 Montrouge, France. Writing: review and editing; read and approved the final manuscript.; m Mathieu A. Derbanne Dentist, Service de Santé des Armées, Direction Inter Armée du Service de Santé, Forces Armées en Guyane, ADDA Member, Cayenne, French Guiana, France. Concept and design; writing: review and editing; read and approved the final manuscript.; n Philippe François Associate Professor, Department of Biomaterials, UMR 1333 Oral Health, University of Paris Cité, Bretonneau Hospital, Assistance Publique–Hôpitaux de Paris (AP-HP), 1 rue Maurice Arnoux, 92120 Montrouge, France. Concept and design; acquisition, analysis, and interpretation of data; writing: original draft preparation; writing: review and editing; read and approved the final manuscript.

**Keywords:** adhesion, biomaterials, bond strength, dental adhesive, education

## Abstract

**Purpose:**

To evaluate whether single-day, gamified training (called “Battle of the Bonds”) improves operators’ immediate macro dentin shear bond strength (SBS) values and perceived learning in undergraduate and continuing dental education about dental adhesives, composite resin, and light-curing units.

**Materials and Methods:**

In July 2025, 14 dentists and 21 final-year students performed four macro-SBS tests before and eleven after lectures on adhesive systems, composite selection, and light-curing. SBS was measured on human dentin for each participant’s routine adhesive and representatives of all actual adhesive/generation families (from three-step etch-and-rinse to universal adhesives). Post-course satisfaction was measured using a 13-item Likert questionnaire (ranging from 1 to 5). Paired and independent t-tests were used to compare the SBS (α = 0.05).

**Results:**

The baseline SBS with the participants’ own adhesive was similar between the dentists and the students (12.5 ± 6.4 MPa vs 13.1 ± 6.6 MPa, P = 0.68). After training, the mean SBS with the participants’ own adhesive significantly increased to 18.5 ± 6.3 MPa for the dentists (+67%) and to 17.3 ± 6.0 MPa for the students (+27%) (both P < 0.05). Satisfaction with training and perceived knowledge gains were very high (4.9 ± 0.5 for dentists and 4.9 ± 0.3 for students).

**Conclusions:**

A single-day, competition-based curriculum that couples concise lectures with hands-on SBS testing yields rapid, clinically meaningful improvements and strong engagement across predoctoral and practitioner audiences. This experiential format may help standardize technique-sensitive restorative skills in dental education.

Light-cured dental adhesives and direct composite resins are widely considered the standard of care for direct restorations.^7,14^ Clinical outcomes of bonded composite resin restorations reflect a combination of patient risk, case complexity, materials, and operator-related factors.^11,18,38^ Across studies, the bonding strategy and adherence to protocols (including light-curing) often show stronger associations with performance than the choice of direct composite resin does, whereas the operator technique makes a substantial contribution that interacts with patient and case factors.^11,18^ Accordingly, optimizing adhesive protocols^23^ and their execution through efficient light-curing^24,25^ is at least as important as selecting among contemporary materials.^9,10^ Numerous reviews indicate that durable bonding is technique sensitive and protocol dependent, underscoring the need for strong foundational knowledge and procedural standardization.^21,22^

Given these considerations, there is a clear need for targeted training for practitioners and students, especially because in routine practice, it can take years before the consequences of suboptimal bonding protocols become apparent.^23^ The “Battle of the Bonds”,^3,8^ a program conceived in 2000 by Michel Degrange and inspired by the hands-on update sessions introduced by Jacques Holz, was designed to strengthen both the theoretical understanding and the practical execution of dental adhesives, direct composite resins, and light-curing techniques at a time when adhesive dentistry was rapidly evolving.

Historically, two bonding strategies have been distinguished.^19,41^ Etch-and-rinse (ER) systems use a separate phosphoric acid etch (typically 35–40% H_3_PO_4_) to demineralize enamel and dentin, followed by priming and adhesive application, implemented clinically as three-step ER (ER3: etching-primer-bonding) or two-step ER (ER2: etching-primer+bonding).^19^ Self-etch (SE) systems incorporate acidic functional monomers that simultaneously condition and prime the substrate and are delivered as two-step SE (SE2: self-etch primer-bonding) or one-step SE (SE1: all-in-one).^41^ Selective enamel etching is used to enhance the enamel bond when employing self-etch adhesives. It consists of applying 35–40% phosphoric acid to the enamel margins only, then placing the adhesive on enamel and dentin without prior dentin etching. This hybrid approach, initially adopted with self-etch adhesives and now widely used with universal adhesives, aims to maximize enamel bonding while limiting dentin over-etching.^22^

Universal adhesives, the latest generation of dental adhesives, were introduced to the market in 2011.^22^ A universal dental adhesive is a typically single-bottle bonding system designed for use in etch-and-rinse, self-etch, or selective-enamel-etch strategies. These adhesives also provide adhesion to enamel and dentin as well as to indirect restorative substrates (eg, silica-based ceramics, zirconia, metals, and composite resins) and are compatible with self- and dual-cure resin materials, either intrinsically or via a dedicated activator, in accordance with the manufacturer’s instructions.^22^ Although simplified adhesives are by no means less technique sensitive than other systems^22,28^ are, their performance has advanced to the point that it is no longer defensible to claim that complex, multi-bottle adhesives are inherently superior.^27^

Whereas reliable enamel bonding is relatively straightforward after phosphoric acid etching, dentin bonding is inherently more complex because dentin contains more water and proteins (including matrix metalloproteinases), presents a tubular microstructure, and, after phosphoric acid etching, may exhibit a demineralized zone that is not fully resin infiltrated. These features, and others, explain why most laboratory adhesion studies concentrate on dentin as the critical and more technique-sensitive substrate.^19,22^

The aim of this study was to evaluate the educational value of a structured training program, called “Battle of the Bonds” by its creators,^3,8^ that combines lectures with gamified, competition-based practical sessions. Designed for practicing clinicians (and extended in this publication to final-year dental students to better understand its extended pedagogical interest), the program aims to consolidate knowledge of enamel and dentin bonding mechanisms and refine the clinical use of contemporary adhesive systems through macro-shear bond strength (SBS) testing on dentin, optimize light-curing practices by emphasizing evidence-based parameters (irradiance and exposure time), and inform material selection among families of direct composite resins.

We hypothesized that a single-day standardized training session would not significantly change the immediate dentin shear bond strength of any tested adhesive in student or practitioner cohorts.

## MATERIALS AND METHODS

### Study Design

Thirty-five volunteers participated in the study, including 14 practicing general dentists (12 private practitioners and 2 also affiliated with the university) and 21 dental students in the final phase of their curriculum (between the end of their fifth and sixth years, coming from six different hospitals, and using various dental adhesives). The age distribution within the practitioner group was representative of the general dentist population.

The investigation took place in July 2025 at the Faculty of Dental Surgery of Paris-Cité University. Inclusion in the analysis required completion of every SBS test, participation in all theoretical lectures and submission of the questionnaire response at the end of the formation. All 35 volunteers met these criteria, yielding a final sample of 14 practitioners and 21 students.

At this stage of their studies, students have completed all lectures and practical sessions on adhesion and adhesive application and have been treating patients under faculty supervision since the start of their fourth year, routinely using dental adhesives in conservative dentistry, prosthodontics, and pediatric dentistry.

This structured pedagogical study was conducted over two consecutive days: Day 1 for practicing private dentists invited at no cost and Day 2 for dental students who volunteered for an optional curricular add-on.

### Educational Objectives

The information is delivered in a single-day format combining didactic lectures with interactive, competitive SBS tests.

By the end of the program, participants are expected to be able to do the following:

Deepen their understanding of adhesion mechanisms and distinguish among adhesive system families.
Optimize their dentin bonding protocols to maximize the performance of the adhesives they use in daily practice.
Identify the major families of direct composite resins and evaluate the mechanical, optical, biological, handling, and time-saving advantages of each.
Select a high-quality light-curing unit and apply appropriate polymerization protocols tailored to the restorative techniques they perform.

### Ethical Considerations

This study adhered to the principles outlined in the Declaration of Helsinki and received ethical approval from the AP-HP Centre CER Institutional Review Board (# IRB: IORG0010044). All participants provided written informed consent before the training to participate and to complete the final questionnaire.

### Organization of Training, Specimen Preparation, and Shear Bond Strength Testing

Across the two single-day sessions, each participant performed 15 dentin SBS tests using 11 different adhesives, as detailed below. Table 1 summarizes the materials tested, the abbreviations used in the text, the manufacturers, the standardized protocols applied, and the chemical compositions of the products evaluated in this study.

**Table 1:** Materials, abbreviations, manufacturers, protocol of use, and chemical compositions of the products tested in this study

Type of materials and name	Abbreviation	Manufacturer	Family of adhesive – Number of bottles	Protocol of use for the study	Composition
**Dental adhesive**
Personal adhesive	PA	Unspecified	Unspecified	Unspecified	Unspecified
Optibond FL	OFL	Kerr, Orange, CA, USA	3-step etch-and-rinse (ER3) Adhesive-2 Bottles	3-step etch-and-rinse	Primer: Functional monomers: GPDM; 2-[2-(methacryloyloxy)ethoxycarbonyl]benzoic acid. Base monomer: HEMA. Solvent: ethanol. Adhesive: Base monomers: HEMA; GDMA (2-hydroxy-1,3-propanediyl bismethacrylate). Fillers: glass; ytterbium trifluoride. Silane: 3-trimethoxysilylpropyl methacrylate.
Scotchbond Universal Plus	SBU Plus	Solventum, St Paul, MN, USA	Universal -Adhesive (UA)-1 Bottle	1-step self-etch	Functional monomers: 10-MDP; Vitrebond™ copolymer; HEMA. Solvents: ethanol, water. Filler: non-settling silica. Silane: integrated. Photoinitiator: camphorquinone.
Clearfil Universal Quick 2	CUQ 2	Kuraray, Tokyo, Japan	Universal-Adhesive (UA)-1 Bottle	1-step self-etch 2-step etch-and-rinse 1-step self-etch “optimal protocol”	Single-bottle universal adhesive. Functional monomers: 10-MDP; hydrophilic amide monomer. Base monomer: HEMA. Solvents: ethanol, water. Photoinitiators: camphorquinone; TPO.
Optibond Solo Plus	OSP	Kerr, Orange, CA, USA	2-step etch-and-rinse (ER2)-1 Bottle	2-step etch-and-rinse	Base monomers: HEMA; GDMA. Solvent: ethanol. Fillers: fumed silica; alkali fluorosilicates (Na).
G-Bond	GB	GC Corporation, Tokyo, Japan	1-step self-etch (SE1)-1 Bottle	1-step self-etch	Functional monomer: phosphoric acid ester monomer. Base monomers: UDMA; dimethacrylates. Solvent: acetone Filler: silanated colloidal silica. Photoinitiator present.
G2 Bond Universal	G2B	GC Corporation, Tokyo, Japan	Universal Adhesive (UA)-2 Bottles	3-step etch-and-rinse	Functional monomers: 4-MET; 10-MDP; MDTP Resin matrix: dimethacrylates. Additives: stabilizer (BHT); photo-initiators.
Clearfil SE Bond 2	CSE2	Kuraray, Tokyo, Japan	2-step self-etch (SE2)-2 Bottles	2-step self-etch	Primer: Functional monomer: 10-MDP. Base monomers: HEMA; hydrophilic aliphatic dimethacrylate. Solvent: water. Photoinitiator present. Bond: Functional monomer: 10-MDP. Base monomers: Bis-GMA; HEMA; hydrophobic aliphatic dimethacrylate. Filler: colloidal silica. Photoinitiator.
Universal Bond II	UB II	Tokuyama, Tokyo, Japan	Universal Adhesive (UA)-2 Bottles	1-step self-etch	Liquid A: Functional monomers: phosphoric acid monomer (3D-SR); MTU-6 (thiouracil monomer). Base monomers: HEMA; Bis-GMA; TEGDMA. Solvent: acetone. Liquid B: Silane coupling agent; peroxide/borate redox catalysts. Solvents: acetone, ethanol, water.
Etchant					
K-Etchant syringe	KE	Kuraray, Tokyo, Japan	–	–	Phosphoric acid etching gel (35% H₃PO₄) with colloidal silica (thickener); colorants.
Light cure viscous direct composite resin					
Clearfil Majesty ES-2 (A2)	ES II	Kuraray, Tokyo, Japan	–	–	Resin matrix: Bis-GMA; hydrophobic aromatic and aliphatic dimethacrylates. Fillers: silanated barium-glass; pre-polymerized organic filler. Initiators/additives: camphorquinone; accelerators; pigments.
LED lamp					
Valo Grand cordless	VG	Ultradent, South Jordan, UT, USA	–	–	–
Abbreviations: Bis-GMA: Bisphenol-A diglycidyl dimethacrylate; CQ: Camphorquinone; ER2: 2-step etch-and-rinse adhesive; ER3: 3-step etch-and-rinse adhesive; GDMA: Glyceryl dimethacrylate (2-hydroxy-1,3-propanediyl bismethacrylate); GPDM: Glycerol phosphate dimethacrylate; HEMA: 2-Hydroxyethyl methacrylate; MDP: 10-Methacryloyloxydecyl dihydrogen phosphate; MDTP: 10-Methacryloyloxydecyl dihydrogen thiophosphate; MTU-6: 6-Methacryloyloxyhexyl 2-thiouracil-5-carboxylate; SE1: 1-step self-etch adhesive; SE2: 2-step self-etch adhesive; TEGDMA: Triethylene glycol dimethacrylate; TPO: Phenyl-bis(2,4,6-trimethylbenzoyl)-phosphine oxide; UA: Universal adhesive; UDMA: Urethane dimethacrylate; 4-MET: 4-Methacryloyloxyethyl trimellitate (trimellitic acid ester).

**Fig 1:**
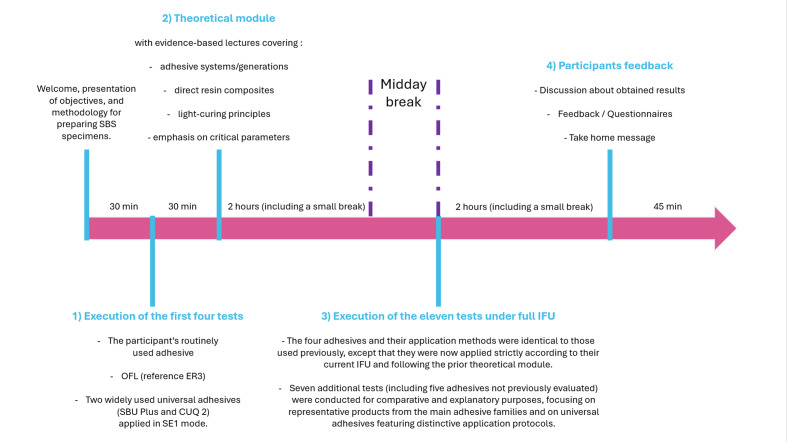
Graphical illustration of the schedule of the training day, highlighting the main objectives and timing of each component.

**Fig 2:**
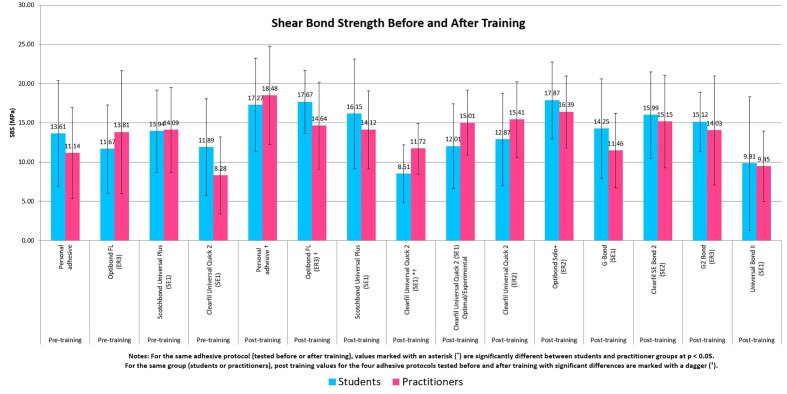
Graphical summary and comparison of shear bond strength across adhesive protocols, pre- and post-theoretical training for students and practitioners.

The training program, synthesized in Figure 1, comprised four sequential components:

The initial practical assessment included four SBS trials: one using each participant’s routine adhesive, one using Optibond FL as the reference ER3, and two using commonly employed universal adhesives in SE1 mode (SBU Plus and Clearfil Universal Quick 2). These tests were carried out without consulting the instructions for use (IFU), in order to evaluate participants’ baseline knowledge of their own adhesive and of key representative systems (OFL as the gold standard and universal adhesives in SE1 mode reflecting their predominant clinical use). For these three standardized tests, only the adhesive family/generation and general handling conditions were disclosed to the participants.
The theoretical module, based on evidence-based lectures, covered adhesive systems and generations, direct composite resins, and light-curing principles, with emphasis on key parameters, scientific interpretation of instructions for use, and practical guidelines to optimize bonding and restorative procedures.
After the midday break, the post-training practical assessment consisted of 11 standardized trials conducted under full IFU. These included all adhesives tested before the theoretical module, as well as benchmark systems representing SE1 (G-Bond), SE2 (Clearfil SE Bond 2), and ER2 (Optibond Solo Plus). Additional universal adhesives with distinctive features were also tested, such as a self-cure setting (Universal Bond II in SE1 mode), a separated primer and adhesive system (G2 Bond in ER3 mode to be compared with the non-containing 10-Methacryloyloxydecyl dihydrogen phosphate Optibond FL), and an ultrasimplified application mode eliminating contact/wait time and reducing air-drying (Clearfil Universal Quick 2). For the latter adhesive, which had already been tested in SE1 mode during the morning session, two additional protocols were included to better understand the potential limitations of its simplified IFU: an ER2 protocol and an “optimal/experimental” condition, in which the adhesive was intentionally applied outside the simplified instructions, using active brushing for 20 s followed by air-drying for 20 s before light-curing (Fig 2).
Immediately after the practical assessments, the participants completed a structured questionnaire on perceived learning gains, satisfaction, and course methodology, followed by a group debrief and a 15-min closing summary of key take-home messages.

To conduct these tests over two consecutive training days, 200 freshly extracted human molars were collected, cleaned of soft tissue, and stored at 4°C in a 1% chloramine solution. SBS test samples were prepared during the week preceding the sessions. The inclusion criterion was the absence of cracks and caries. The roots were shortened using 80-grit abrasive paper on a polishing machine with abundant irrigation. The occlusal enamel was then removed to expose a flat mid-coronal dentin surface (≥ 7 mm^2^) by grinding with 800-grit water-cooled abrasive paper. The residual crowns were embedded in self-curing acrylic resin within plastic cylinders, leaving the flat dentin surface exposed. Each surface was examined at 40× magnification to confirm complete enamel removal and a dentin surface free of debris. The cylinders had parallel top and bottom surfaces, precisely perpendicular to the outer surface, ensuring correct alignment and load distribution during shear bond testing. The resulting SBS dentin samples were stored in distilled water at 37°C until the training sessions.

Teeth were randomly assigned to participants for immediate SBS testing. On each sample, the adhesive was applied either according to the participant’s usual clinical practice (morning session) or according to the manufacturer’s instructions or the specified experimental protocol (afternoon session) and then light-cured with a polywave LED curing unit (VALO Grand Cordless, Ultradent, South Jordan, UT, USA). A cylindrical Teflon mold (height 3 mm; diameter 3 mm) was positioned to build the direct composite resin button in two increments of direct composite resin (Clearfil Majesty ES-2, Kuraray). After polymerization, the mold was removed, and the specimens were visually inspected to detect any major fabrication defects. Any excess composite resin was carefully trimmed with a scalpel. The specimens were then stored in distilled water for a few minutes and tested as soon as possible thereafter to minimize potential bias.

SBS was measured using a universal testing machine (LRX; Lloyd Instruments, Fareham, UK). Shear loading was applied at the direct composite resin‒dentin interface with a chisel-shaped blade aligned parallel to the dentin surface at a crosshead speed of 0.5 mm/min.

Failure modes were not recorded, in keeping with the time constraints of an educational, hands-on *in vitro* design. The samples that did not exhibit visually cohesive failure within the dentin were reprepared and reused. The bonding surface was refreshed by water-cooled grinding with 800-grit silicon-carbide paper to re-expose flat mid-coronal dentin, ensuring complete removal of residual adhesive/direct composite resin. Repreparation was limited to a maximum of five cycles per tooth and permitted only if the pulp chamber remained unexposed and if no cracks or craze lines were observed. After being rinsed and gently air-dried, eligible samples were randomly reassigned to test conditions. Samples showing cohesive dentin fracture or pulp chamber exposure were excluded from further use. During each cycle, all the samples were stored in distilled water at 37°C.

### Assessment Questionnaires

A post-course satisfaction questionnaire was created to assess participants’ perceptions of their baseline knowledge, learning experience, and overall satisfaction with the training. It consists of 13 items rated on a 5-point Likert scale (1 = strongly disagree, 2 = disagree, 3 = neither agree nor disagree, 4 = agree, 5 = strongly agree), with 3 denoting a neutral response. All the items were jointly drafted by the coauthors and reviewed by a methodologist to ensure clarity, consistency, and alignment with the educational objectives. Responses were anonymized using unique participant codes and stored in a password-protected spreadsheet. The questionnaire is presented in Table 3.

**Table 3:** Participant perceptions of their initial knowledge, learning experience, and satisfaction

Participant feedback questionnaire items	Student group (Mean ± SD)	Practitioner group (Mean ± SD)
1) Before the training, I was familiar with the IFU of the direct restorative products, adhesives/composite resin, that I use in daily practice.	3.24 ± 1.00*	3.93 ± 1.00*
2) In the first four knowledge-based SBS tests, I had a reasonably good understanding of the general use of the different adhesive families to achieve good bonding.	3.43 ± 1.03	3.71 ± 1.07
3) The duration of the training (theory and hands-on) was optimal to address the stated topic of optimizing adhesive procedures in direct restorations.	4.33 ± 0.91	4.50 ± 0.65
4) The “competitive game” aspect of the training motivated me to stay attentive.	4.62 ± 0.91	4.57 ± 0.65
5) My understanding of bonding mechanisms improved after this training.	4.81 ± 0.40	4.50 ± 0.65
6) My understanding of the clinical indications for the different families of direct composite resins improved after this training.	4.67 ± 0.66	4.50 ± 0.65
7) I will pay closer attention to the IFU of my direct restorative products (adhesives/composite resins) after this training.	4.81 ± 0.40*	4.29 ± 0.83*
8) I will change the clinical application procedure for my routine adhesive or direct composite resin after this training.	4.43 ± 1.03	4.00 ± 0.88
9) I will change the brand/reference of direct composite resin I use after this training.	3.71 ± 1.19*	2.79 ± 1.05*
10) I will change the brand/reference of curing light after this training.	4.19 ± 1.12*	3.21 ± 1.48*
11) I will change the brand/reference of adhesive after this training.	3.43 ± 1.21	3.00 ± 1.11
12) This training should be a requirement in a practitioner’s continuing education.	4.81 ± 0.40	4.64 ± 0.63
13) Overall, I am satisfied with having participated in this training.	4.90 ± 0.30	4.86 ± 0.53
Abbreviations: IFU: instructions for use, SBS: shear bond strength, SD: standard deviation.Notes: Values with an asterisk are significantly different between student and practitioner groups at P < 0.05.

### Statistical Analysis

Normality of the SBS data (and of pre–post difference scores for paired comparisons) was assessed with the Shapiro‒Wilk test, and the homogeneity of variances was evaluated with Levene’s test. SBS values are reported as the means and standard deviations. Between‐group differences (practicing dentists vs dental students) were tested with independent‐samples t tests at baseline and after training for each of the four adhesives. Within-group changes (pre vs post) were tested with paired t tests, separately for dentists and students, for each adhesive. Two-tailed P values < 0.05 were considered to indicate statistical significance. Statistical analyses were conducted with R (version 4.5.1; R Foundation for Statistical Computing, Vienna, Austria).

## RESULTS

Table 2 summarizes the immediate shear bond strength values obtained before and after training for both students and practitioners across the different adhesive protocols.

**Table 2:** Means and standard deviations of the SBS tests for the various adhesive protocols tested before and after theoretical training

Adhesive protocol	Immediate SBS ± SD for students’ group (in MPa)	Immediate SBS ± SD for practitioner’s group (in MPa)
**Pre-training**		
Personal adhesive – Unknown	13.61 ± 6.76	11.14 ± 5.80
OFL – ER3	11.67 ± 5.63	13.81 ± 7.85
SBU Plus – SE1	13.94 ± 5.25	14.09 ± 5.40
CUQ 2 – SE1	11.89 ± 6.19	8.28 ± 4.92
**Post-training**
Personal adhesive – Unknown	17.27^†^ ± 5.98	18.48† ± 6.30
OFL – ER3	17.67^†^ ± 4.02	14.64 ± 5.53
SBU Plus – SE1	16.15 ± 7.01	14.12 ± 4.96
CUQ 2 – SE1	8.51*^†^ ± 3.71	11.72*^†^ ± 3.24
**Additional groups/adhesive post-training**
CUQ 2 – SE1 “Optimal/Experimental Protocol”	12.01 ± 5.41	15.01 ± 4.15
CUQ 2 – ER2	12.87 ± 5.90	15.41 ± 4.80
OSP – ER2	17.87 ± 4.92	16.39 ± 4.59
GB – SE1	14.25 ± 6.37	11.46 ± 4.75
CS2 – SE2	15.99 ± 5.51	15.15 ± 5.91
G2B – ER3	15.12 ± 3.79	14.03 ± 6.94
UB II – SE1	9.81 ± 8.52	9.45 ± 4.49
Notes: For the same adhesive protocol (tested before or after training), values marked with an asterisk (*) are significantly different between students and practitioner groups at P < 0.05.For the same group (students or practitioners), post-training values for the four adhesive protocols tested before and after training with significant differences are marked with a dagger (^†^).

### Initial Tests (Pretraining)

No significant differences in immediate SBS were observed between cohorts across the four shared protocols (students vs practitioners), namely, personal adhesive 13.61 ± 6.76 MPa vs 11.14 ± 5.80 MPa, Optibond FL (ER3) 11.67 ± 5.63 MPa vs 13.81 ± 7.85 MPa, Scotchbond Universal Plus (SE1) 13.94 ± 5.25 MPa vs 14.09 ± 5.40 MPa, and Clearfil Universal Quick 2 (SE1) 11.89 ± 6.19 MPa vs 8.28 ± 4.92 MPa (all P > 0.05).

### Post-training Tests (Protocols Assessed Both Pre- and Post-training)

SBS increased significantly with the personal adhesive for students (13.61 ± 6.76 MPa to 17.27 ± 5.98 MPa, P < 0.05) and practitioners (11.14 ± 5.80 MPa to 18.48 ± 6.30 MPa, P < 0.05). With respect to Optibond FL (ER3), the SBS values improved significantly for students (11.67 ± 5.63 MPa to 17.67 ± 4.02 MPa, P < 0.05) but not for practitioners (13.81 ± 7.85 MPa to 14.64 ± 5.53 MPa, P > 0.05). With respect to Clearfil Universal Quick 2 (SE1), the students’ SBS values decreased (11.89 ± 6.19 to 8.51 ± 3.71, P < 0.05), whereas the practitioners’ SBS values increased (8.28 ± 4.92 MPa to 11.72 ± 3.24, P < 0.05).

### Post-training Tests (Additional Tests)

Among the additional post-training protocols, no significant differences in immediate SBS were observed between cohorts. Optibond Solo Plus (ER2) yielded the highest immediate SBS for students 17.87 ± 4.92 MPa vs 16.39 ± 4.59 MPa for practitioners. For Clearfil Universal Quick 2, the ER2 protocol 12.87 ± 5.90 MPa for students vs 15.41 ± 4.80 MPa for practitioners) and the “optimal/experimental” SE1 handling 12.01 ± 5.41 MPa for students vs 15.01 ± 4.15 MPa for practitioners were recorded. Universal Bond II (SE1) showed the lowest values, 9.81 ± 8.52 MPa for students and 9.45 ± 4.49 MPa for practitioners.

### Post-course Satisfaction

Ratings were highly favorable (Table 3). The competitive format was endorsed (4.62 ± 0.91 for students and 4.57 ± 0.65 for practitioners), and perceived learning was high (4.81 ± 0.40 for students and 4.50 ± 0.65 for practitioners). Practitioners reported significantly greater perceived baseline IFU knowledge (3.93 ± 1.00 for practitioners vs 3.24 ± 1.00 for students, P < 0.05), whereas students more often intended to know the IFU of their products post-course (4.81 ± 0.40 for students vs 4.29 ± 0.83 for practitioners, P < 0.05).

## DISCUSSION

The null hypothesis was rejected: the single-day standardized training session significantly changed the immediate dentin shear bond strength in both cohorts, with the most notable increase observed when participants used their personal adhesives, in both student and practitioner groups.

This two-day blended intervention, combining didactic teaching with standardized, competitive macro-SBS testing, produced measurable, adhesive- and cohort-dependent changes in immediate bond strength. To accommodate the educational format, the SBS component necessarily departed from conventional laboratory practice: teeth were reprepared and reused (up to five times) provided that the pulp chamber remained unexposed, and failure modes were not recorded because of the lack of time available during the training. These procedural choices diverge from ISO 29022:2013 recommendations but are defensible in an instructional setting focused on rapid, objective feedback.^15^ In parallel, it is well established that bonding to coronal dentin exceeds bonding to deeper dentin, largely because the tubule density and diameter increase with depth, increasing dentin permeability and water content.^20^ Thus, it could be expected that as the samples were refreshed throughout the day, progressively deeper dentin likely reduced the absolute obtained SBS values. Accordingly, each session was initiated with intact teeth and specimens were replaced only when cohesive fractures occurred during testing or when the pulp chamber became visible by transillumination. As a result, initial practical assessments were conducted, on average, on higher‑quality dentin than post-training practical assessment, which likely lowered absolute SBS values as the day progressed. This conservative bias would be expected to attenuate training‑related improvements rather than inflate them, suggesting that the observed significant differences are, if anything, lower‑bound estimates of the true effect.

Despite this possible substrate trend, the most consistent and statistically significant improvement occurred with each participant’s personal adhesive in both cohorts (from 13.61 ± 6.76 to 17.27 ± 5.98 MPa for students and from 11.14 ± 5.80 to 18.48 ± 6.30 MPa for practitioners). This corresponded to +26.89% and +65.89% increases in SBS values for students and practitioners, respectively, in the routing of dental adhesive use, even though the first trial in both cohorts, performed pretraining with each participant’s personal adhesive, was conducted on a previously untested “ideal” dentin substrate. With regard to the brands used by participants, students applied three different formulations selected by their respective hospitals (two universal adhesives and one ER2), whereas practitioners used a much wider range of products, with a clear predominance of universal adhesives. In this context, what matters is not the absolute value obtained with each personal adhesive, but rather the statistically significant progress achieved by each participant using the same material before and after the training. This confirms that the improvement observed is primarily related to the educational effect and proper application of the instructions for use, rather than to differences in adhesive brands. This confirms that the improvement observed is primarily related to the educational effect and proper application of the instructions for use, rather than to differences in adhesive brands, thereby reinforcing the idea that the training itself was the main driver of performance enhancement. This finding supports the interpretation that a short, structured refresher coupled with immediate, objective feedback can sharpen the execution of familiar protocols.^29,35^ The greater post-training gain observed among practitioners with their personal adhesive is psychologically plausible. Over time, routine clinical pressures foster automaticity and entrenched habits that can drift from the IFU of dental products. This phenomenon is also called normalization of deviance.^2^ Targeted, feedback-rich practices likely deautomatize these routines, enabling rapid correction of suboptimal microsteps.^16^ Training with the exact material used in daily work also leverages specificity of practice, facilitating near-transfer compared with brand- or protocol-switching. Finally, procedural skill decay between continuing-education episodes may accentuate the headroom for improvement among experienced clinicians. Together, these mechanisms explain why the greatest improvement emerged when practitioners retested their own adhesive under coached, IFU-aligned conditions.^36^ Moreover, the literature cautions that the predictive value of immediate laboratory bond tests for long-term clinical performance is limited or inconsistent; thus, the improvements observed here should be interpreted as proxies for better technique and protocol adherence rather than direct guarantees of clinical longevity.^13,40^ Although *in vitro* bond strength tests must be interpreted cautiously, given the experimental sources of bias, such as adhesive mechanical properties, interface solicitation and film thickness,^30,33,34^ the within-formulation gains observed here most plausibly reflect improved protocol execution rather than material differences. Such procedural improvements are expected to strongly favor clinical performance. Overall, these findings are consistent with the state-of-the-art consensus that durable bonding is technique sensitive and protocol dependent, with outcomes driven by etching strategy selection, active application with adequate solvent evaporation, and appropriate light-curing with a polywave LED lamp: some key messages given during training.^6,12,25,37^

From an educational standpoint, using a multistep ER3 adhesive was essential for revisiting, step by step, the adhesion sequence (etching–priming–bonding) and consolidating procedural knowledge through guided practice.^31^ With this adhesive, the bond strength increased significantly among the students from 11.67 ± 5.63 to 17.67 ± 4.02 MPa, whereas the bond strength among the practitioners did not significantly increase from 13.81 ± 7.85 to 14.64 ± 5.53 MPa. This divergence likely comes from differences in baseline training and ingrained clinical habits: for years, the dental curriculum at Paris-Cité University has emphasized simplified universal adhesive systems, whereas the more complex multibottle approach of Optibond FL is no longer used in daily practice. Students who reported lower precourse familiarity with manufacturer instructions (Table 3) (mean score 3.24 ± 1.00 vs practitioners’ 3.93 ± 1.00; P < 0.05) had more room for procedural learning of a family of adhesives that most of them had never used before.^31^ Conversely, experienced practitioners, who were already comfortable and may have been trained previously with multistep adhesives and who were more aware of IFU than students were, were less responsive to a refresher and applied their routine habits.^31,32^ Both cohorts perceived clear learning gains regarding bonding principles (4.81 ± 0.40 for students and 4.50 ± 0.65 for practitioners; P > 0.05). After the course, students more often indicated that they would pay closer attention to IFU than practitioners who were more accustomed to it before the training (4.81 ± 0.40 for students and 4.29 ± 0.83 for practitioners; P < 0.05).

With IFU settings, Clearfil Universal Bond Quick 2 (CUQ2) underperformed in its standard SE1 mode, with student performance declining from 11.89 ± 6.19 to 8.51 ± 3.71 MPa after training, whereas practitioners’ performance improved from 8.28 ± 4.92 to 11.72 ± 3.24 MPa. The “optimal/experimental” SE1 handling using gold-standard application for SE1 adhesive led to higher SBS values for both groups (12.01 ± 5.41 MPa for students and 15.01 ± 4.15 MPa for practitioners). These patterns mirror evidence that active application (scrubbing) and adequate solvent evaporation/air-drying can enhance the infiltration and dentin bonding of simplified or one-bottle systems.^1^ Universal Bond II (self-curing, two-bottle) also yielded low immediate SBS in post-lecture protocols (9.81 ± 8.52 MPa for students and 9.45 ± 4.49 MPa for practitioners). A plausible mechanism is its complex self-curing chemistry, with polymerization kinetics that differ from those of light-activated UAs; but it also has weak adhesive properties when it is used in the SE1 mode.^26^ These pedagogical examples increased participants’ awareness of the need for training in innovation,^32^ the importance of “formulation-dependent” adhesive performance and the fact that any simplification proposed does not always translate into equal performance achieved.^5,6,12,37^

Beyond the objective outcomes, participants rated the learning experience extremely positively, with overall satisfaction near the ceiling in both groups (4.90 ± 0.30 for students and 4.86 ± 0.53 for practitioners, P > 0.05). These data indicate that the gamified, feedback-rich format clearly enhances skill learning and adaptability, which are crucial for technique-sensitive tasks such as adhesive handling. Pedagogically, this aligns with evidence that gamification can enhance engagement and learning outcomes in health profession education when meaningful challenges and immediate scoring/feedback are used.^4,39^

This study clearly has several limitations. First, the primary outcome, immediate macro-shear bond strength, is an educational proxy rather than a clinical endpoint and has been measured through simplifications in comparison to *in vitro* studies.^13^ Second, by design, the evaluation focused on levels 1 and 2 of Kirkpatrick’s framework (satisfaction and skills development) and did not assess levels 3 or 4 (behavior change in practice and patient outcomes).^17^ Accordingly, the present findings should be interpreted as evidence of short-term learning gains and acceptability rather than definitive impacts on long-term clinical performance.

This single-day, gamified program called “Battle of the bonds” and integrating evidence-based lectures and standardized hands-on exercises improved immediate dentin bonding performance and heightened attention to adhesive dentistry, efficient light-curing and composite resin choice across students and practitioners. High levels of satisfaction and perceived learning support the inclusion of this brief, feedback-rich format in undergraduate curricula and continuing professional development.

### Acknowledgments

The authors would like to thank: Michel Degrange, Jacques Holz and Bernard Lapostolle for creating this format; the ADDA (Académie De Dentisterie Adhésive) for allowing this training course to be evaluated; and Valentine Orgueil for organizing the event.

#### Disclosure

Emmanuel Bourdageau is an employee of the French division of Kuraray. The other authors of this publication declare that they have no conflicts of interest in connection with this publication.

#### Clinical relevance

This gamified, hands-on training enhances adhesive dentistry skills by providing practitioners with a valuable refresher while offering final-year students a practical consolidation of their knowledge. Such a format supports both continuing education and undergraduate preparation for clinical practice.
